# In danger: HIV vaccine research and development in Europe

**DOI:** 10.1371/journal.pgph.0004364

**Published:** 2025-04-08

**Authors:** Roger Tatoud, Yves Lévy, Roger Le Grand, Jose Alcami, Giorgio Barbareschi, Christian Brander, Andrea Cara, Behazine Combadière, François Dabis, Sarah Fidler, Tomáš Hanke, Carolina Herrera, Gunilla B. Karlsson Hedestam, Hester Kuipers, Sheena McCormack, Christiane Moog, Giuseppe Pantaleo, Laura Richert, Rogier W. Sanders, Robin Shattock, Hendrik Streeck, Rodolphe Thiebaut, Alexandra Trkola, Klaus Üeberla, Marit J. Van Gills, Ralf Wagner, Winfried Weissenhorn, Yazdan Yazdanpanah, Gabriella Scarlatti, Jean Daniel Lelièvre

**Affiliations:** 1 Origena Consulting, Ferney Voltaire, France; 2 Vaccine Research Institute, Créteil, France; 3 Department of Infectious Diseases Models for Innovative Therapies, CEA, Fontenay aux Roses, France; 4 Fundació de Recerca Clínic Barcelona-Institut d’Investigacions Biomèdiques August Pi i Sunyer, Barcelona, Spain; 5 European AIDS Treatment Group, Brussels, Belgium; 6 IRSICAIXA AIDS Research Institute, Barcelona, Spain; 7 Istituto Superiore di Sanità, Rome, Italy; 8 Sanofi, Marcy-l’Étoile, France; 9 University of Bordeaux, Bordeaux, France; 10 Department of Infectious Disease, Imperial College London, London, United Kingdom; 11 The Jenner Institute, University of Oxford, Oxford, United Kingdom; 12 CONRAD, Macon & Joan Brock Virginia Health Sciences at Old Dominion University, Norfolk, Virginia, United States of America; 13 Department of Microbiology, Tumor and Cell Biology, Karolinska Institute, Stockholm, Sweden; 14 IAVI, Amsterdam, The Netherlands; 15 MRC Clinical Trials Unit, University College London, London, United Kingdom; 16 UMR_S INSERM, Université de Strasbourg, Strasbourg, France; 17 Department of Medicine and Laboratory Medicine, University of Lausanne, Lausanne, Switzerland; 18 Amsterdam University Medical Centers, Amsterdam, Netherlands; 19 Department of Medicine, Imperial College London, London, United Kingdom; 20 Institute of Virology, Bonn, Germany; 21 Institute of Medical Virology, Zurich, Switzerland; 22 Virology Institute, Erlangen, Germany; 23 Department of Medical Microbiology and Infection Prevention, University of Amsterdam, Amsterdam, The Netherlands; 24 Institute of Medical Microbiology and Hygiene, Molecular Microbiology (Virology), Regensburg, Germany; 25 Institut de Biologie Structurale (IBS), Grenoble, France; 26 ANRS Emerging Infectious Diseases, Paris, France; 27 Viral Evolution and Transmission Group, IRCCS Ospedale San Raffaele, Milan, Italy; 28 Department of Infectious Diseases & Clinical Immunology, Henri Mondor University Hospital, Créteil, France; Universidade Catolica Portuguesa, PORTUGAL

## Abstract

Highly effective antiretroviral-based HIV prevention plays an important role in ending the global HIV/AIDS epidemic. However, the sustainable control of the epidemic is hampered by unequal access to prevention options, including HIV testing, alongside with drug resistance and ongoing barriers to accessing sustainable HIV treatment. Therefore, an HIV vaccine, combined with effective prevention and treatment, remains an absolute necessity to control the epidemic. Yet, the recent discontinuation of four major vaccine efficacy studies is raising concerns about the future of HIV vaccine research and development globally, and particularly in the European region where funding for vaccine research and development has shrinked. This viewpoint emphasises that supporting HIV vaccine research and development at the European level remains crucial: it is not only necessary to control the epidemic, but it promotes innovation, strengthens health security, epidemic preparedness, and health sovereignty while contributing to the economies of European nations.

## Introduction

While HIV prevention and treatment have made great strides toward reducing the number of HIV-related deaths and new HIV acquisitions, HIV continues to threaten and burden public health systems globally. An HIV vaccine remains essential for reaching the 95-95-95 targets set by UNAIDS and for the durable control of the epidemic. However, despite significant scientific advances and the pursuit of diverse vaccine development strategies [1,2], the disappointing results of recent prophylactic HIV vaccine efficacy trials are causing uncertainty about the research and funding trajectory of HIV vaccine research and development (R&D) globally. This is particularly salient for the European region with its research ecosystem structured around, and dependent on, national and supranational collaborations and research strategies. With competing global health priorities and limited prospects for new funding opportunities, sustaining and optimising prophylactic HIV vaccine R&D at the European level is vital as it not only fosters innovation, but also yields global social and economic benefits that extend beyond the creation of an HIV vaccine. We believe that given its long-standing legacy in vaccine development, innovation, and academic excellence, Europe holds a crucial position in the pursuit of an HIV vaccine and that investing in vaccine R&D is an opportunity for a comprehensive engagement in a multi-sectoral endeavour with substantial societal and economic benefits for Europe and beyond.

### HIV remains a public health challenge across Europe

Effective treatment and pre-exposure prophylaxis (PrEP) have enabled rapid progress in controlling the epidemic and HIV is nowadays deceptively viewed as a manageable chronic condition, when treatment is available, and for which there is effective prevention. However, equitable access to prevention and treatment remains a significant challenge in many settings, including in the World Health Organization (WHO) European Region (Box 1). HIV testing, the gateway to healthcare, remains a concern with late HIV diagnosis a persistent challenge in most European countries. Stigma and discrimination continue to hamper the required expansion of treatment and prevention [3] and equitable access to current and next generation PrEP remains problematic [4–6]. Developments in the USA in 2025 serve as a stark reminder that our reliance on antiretroviral therapy (ART) alone weakens the HIV response and poses a significant risk to the goal of ending the HIV/AIDS epidemic. [7]

**BOX 1. HIV in the WHO European Region**

According to WHO and the European Centre for Disease Prevention and Control, there were 112,883 newly reported HIV acquisitions across 47 of 53 European countries in 2023, with 24,731 in European Union (EU) and European Economic Area (EEA) countries. The crude incidence of new HIV diagnoses in the region was 12.7 per 100,000 population, varying by sub-regions, with higher rates in Eastern Europe and lower rates in Central Europe. A total of 7,878 individuals were diagnosed with AIDS in 41 countries of the WHO European Region, resulting in a rate of 1.2 new diagnoses per 100,000 population [38]. Most newly diagnosed people were from the eastern part of the region, while countries in the EU/EAA mostly showed a decrease in the rates of new diagnoses. Reporting has been delayed by the COVID-19 pandemic suggesting that current data may be underestimated.

In 2022, according to WHO, only 71% of people living with HIV in the European region knew their HIV status, 87% of people with diagnosed HIV received antiretroviral treatment (ART), and 90% of people receiving ART had viral suppression.

Access to PrEP remains limited in the region, with an estimated 68,089 oral PrEP initiations in 2020 [39]. PrEP rollout varies from country to country and is hindered by many challenges, including access to services, cost, limited awareness, stigma, and lack of implementation projects [40].

Annual treatment costs vary widely among countries in the region. Data reported by 18 EU/EEA and non-EU/EEA countries in 2014 and 20 EU/EEA and non-EEA countries in 2016 highlights the variation in the cost of antiretroviral drugs between countries. The cost of drugs per person per year ranged from EUR 3,800 to EUR 17,500 in 2014 and from EUR 1,000 to more than EUR 20,000 in 2016 [41]. Despite recent improvements in the HIV response across the WHO European region, significant disparities and inequalities persist. Urgent action is required to tackle challenges such as undiagnosed HIV cases, late presentation, stigma, discrimination, and barriers to accessing care [42].

Overall, progress in controlling the HIV/AIDS epidemic in Europe remains fragile and threatened by pandemic, natural disasters, armed conflicts, and migration [8]. The ongoing conflict in Ukraine disrupted the progress made in tackling HIV in the Eastern European region and is a stark reminder of the vulnerability of some public health systems and the need for solutions adapted to specific settings. The COVID-19 pandemic has demonstrated that viruses can cross national borders, emphasising that while the scale of the HIV/AIDS epidemic in Europe may not match that of the hardest-hit nations in eastern and southern Africa, effective epidemic control requires a global approach. It has also illustrated that new virus variants can potentially evolve in immunocompromised individuals such as untreated people living with HIV [9]. The European Commission (EC) has acknowledged that gaining control over an epidemic in areas where it is most severe will aid in controlling it in other regions. While increasing access to PrEP in the European Region is paramount, the current situation and prospects warrant the continued development of an HIV vaccine to strengthen existing HIV prevention and treatment strategies.

### New perspectives for HIV vaccine R&D

Vaccination is considered the second most effective public health intervention available after access to safe water [10]. It is estimated that every US dollar invested in global vaccination programmes saves USD 21 in healthcare costs, lost wages and lost productivity due to illness and death [11]. For example, COVID-19 vaccines have without doubt changed the course of the COVID-19 pandemic and saved tens of millions of lives globally [12]. Although designing a safe and effective HIV vaccine is considerably more challenging [13], a vaccine that enables broad and durable protection would be an important public health intervention, especially if combined with other effective treatment and prevention interventions [14]. Modelling studies show that even a partially effective HIV vaccine would help control the epidemic [15]. In addition, an HIV vaccine could be less stigmatising than taking antiviral drugs on a regular basis [16]. By preventing HIV acquisition an HIV vaccine also comes with the collateral benefit of reducing the incidence of co-infections, such as tuberculosis, and co-morbidities such as cancer and cardiovascular diseases associated with HIV [17,18].

European consortia and product development partnerships have developed several HIV vaccine candidates, including viral proteins, conserved and mosaic immunogens, as well as various delivery platforms such as DNA, replication-competent and replication-incompetent viruses, many of which have been tested in clinical trials (S1 Table). Present-day immunogens include stabilized trimeric proteins that emulate the native HIV-1 envelope, structure-based immunogens, novel mosaic and conserved antigens, and antibodies targeted at dendritic cells, combined with both existing and new nucleic acid and viral platforms [19], such as mRNA [20]. New clinical testing strategies, such as the experimental medicine approach [21], are expected to accelerate iterative vaccine evaluation. HIV offers a challenging model for developing vaccines and it is imperative to acknowledge and support the technological progress originating from HIV vaccine R&D, as they foster advancements in the broader field of vaccine development.

### Supporting a robust research base in Europe

The EC remains a significant investor in HIV and AIDS prevention, and HIV remains a priority of EC investments in poverty-related diseases R&D. Bolstered by complementary efforts of Member States and European-based philanthropic organisations, the EC committed EUR 220 million under Horizon 2020 [22]. The EU Research Framework programmes (including FP7, Horizon 2020, and the European and Developing Countries Clinical Trials Partnership - EDCTP) play a crucial role in fostering vaccine development. EDCTP, in particular, has stimulated partnerships between Europe and Africa, as well as facilitated the transfer of knowledge and skills. As the Research Framework programmes evolve (into Horizon Europe and EDCTP3), it is essential that HIV vaccine R&D remains part of the funding portfolio.

Over the years, the European-led HIV vaccine research base has demonstrated excellence and innovation with limited EC funding specifically allocated to HIV vaccine R&D and disruptive funding cycles. Capitalizing on previous investments in HIV vaccine R&D is therefore important, as these investments have supported highly competitive research and development activities in Europe. These investments have also significantly contributed to building research capacity and capability, that contributed to the rapid development of COVID-19 vaccines. HIV vaccine R&D may now leverage new investment currently considered in the EU for vaccine research in response to the COVID-19 crisis.

**Fig 1:**
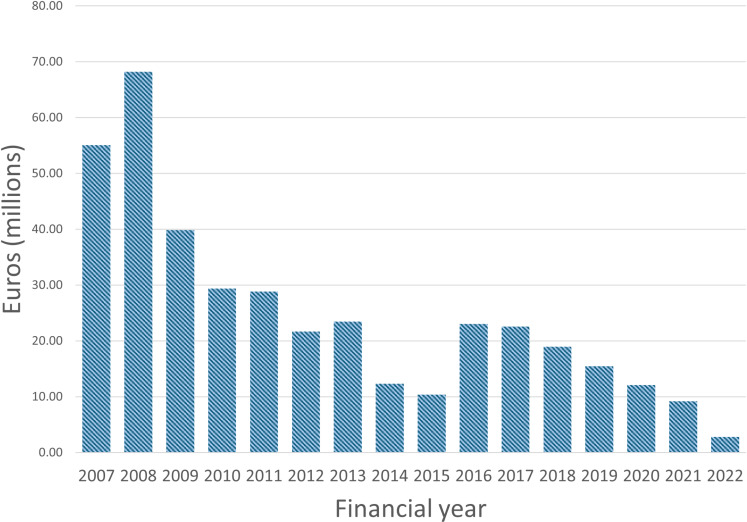
European Union Member States funding dedicated to HIV vaccine research and development (Source: G-Finder[ **24])**.

However, the funding allocated by the EC for HIV vaccine R&D constitutes only a minor portion of the global investment in vaccine R&D [23], and has declined from over EUR 15 million in 2009 to EUR 9 million in 2020 [24] (Fig 1) to, in essence, disappear in Horizon Europe. HIV vaccine R&D requires long-term, sustained efforts to fulfill the promise of ending the HIV/AIDS epidemic. This work spans preclinical research and early- to late-phase human trials, evaluating candidates based on the scientific understanding of what could be an effective vaccine strategy. Significant progress is underway, with broadly neutralising antibodies and other approaches under investigation [25,26]. Notably, EU-led efforts make important contributions to the global HIV vaccine enterprise, which has been dominated by investigators and funders in the United States. In the current context of budgetary constraints and policy shifts worldwide, an autonomous and independent Europe has the potential to accelerate progress and expand research efforts. This will require decades of smart and lasting investment to sustain existing expertise, support new generations of researchers, and signal the EU’s long-term commitment to industry and innovation. In the absence of funding specifically allocated to HIV vaccine R&D and the integration of researchers and infrastructures into a European network supported by a long-term plan, Europe-led HIV vaccine R&D is at risk of disappearing, and Europe of losing its cutting-edge expertise, international competitiveness, and anticipated years of future contribution to R&D.

### Vaccine research is innovative with high economic impact

HIV vaccine research has played a pivotal role in advancing immunogen design strategies, vaccine platforms, innovative study designs, capacity building, community engagement, and collaboration with regulatory authorities to expedite approval processes. Over time, investment in vaccine research, including in Europe, has facilitated the creation of groundbreaking technologies that have played a pivotal role in effectively responding to the COVID-19 pandemic [27,28].

HIV vaccine R&D has driven innovations in basic immunology, structural biology, immunogen design [29]. The advancements made through this research have extended beyond HIV, contributing to the development of other vaccines against infectious diseases such as Lassa virus [30], Nipah virus [31], Ebola virus [32], and addressing health challenges such as cancer and treatments for immune-mediated diseases [33]. These efforts combined with other HIV vaccine-related improvements can strengthen Europe’s ability to respond to current and future pandemics [34]. Moreover, this research will lead to long-term and sustainable economic advantages, further strengthening Europe’s position in combating public health crises. A 2020 report commissioned by the Bill & Melinda Gates Foundation estimated that the economic benefits of a global equitable COVID-19 vaccine alone for 10 donor economies, including four European countries, would be at least USD 153 billion in 2020-21, rising to USD 466 billion by 2025. This is more than 12 times the USD 38 billion estimated total cost of the Access to COVID-19 Tools (ACT) Accelerator [35].

Europe’s leadership in innovation has been demonstrated, and it is crucial to maintain momentum by promoting and supporting innovation that drives economic growth and enhances health security globally.

### Re-engaging stakeholders in HIV vaccine research

The difficulties and costs associated with developing an HIV vaccine, and particularly the cost of conducting efficacy trials, have led academics and the pharmaceutical industry to pivot to research challenges that offer a greater likelihood of success. Industry stakeholders still recognise an enduring need for a safe and effective preventive HIV vaccine. However, there is no longer any large company with an active HIV vaccine programme after the termination of the MOSAICO trial [36]. Some industry players acknowledged that the size of the HIV vaccine market may be underestimated or incorrect due to outdated or incomplete market analyses. In addition, an overestimation of the barriers presented by scientific challenges faced in vaccine R&D further reduces interest to engage in HIV vaccine R&D. Tools must be developed in collaboration with organisations such as WHO – for example, Full Value of Vaccine Assessments (FVVA), Preferred Product Characteristics (PPC), and demand and market forecasts – to quantify and demonstrate the market potential for an HIV vaccine and to assess the unmet health need. Engagement with the industry can be further facilitated through active near-term push-and-pull mechanisms by governments and global health organisations with the support of a strong grassroots advocacy movement.

Remarkably, prophylactic HIV vaccine development is also advanced by smaller biotechnology companies that share with the academic sector the struggle of identifying and securing funding. These biotechnology companies bring innovation forward and have the potential to contribute to the modernisation of various industries. The EC has recognised the value of supporting innovation through the European Innovation Council, which could be an important instrument to support and boost innovative vaccine-related research by Europe-based small and medium enterprises.

In addition, it is essential to inspire a new generation of researchers to participate in vaccine R&D, by providing a variety of funding opportunities to encourage early career investigators to engage on a challenging research path. Likewise, there is a need to generate demand and garner grassroots support for an HIV vaccine through enhanced research literacy. Efforts must be made to engage actively and effectively the community of people most affected by and living with HIV. Their perspectives, experiences, and needs can provide valuable insights and contribute to more effective research and product development strategies. In doing so, it will be important to ensure that an HIV vaccine is developed and integrated into existing and future effective prevention strategies [37].

### Organising research at supranational level

Structuring and strategising HIV vaccine R&D in Europe, as well as preparing for roll-out and access, will require multi-stakeholder engagement, which can be initiated once there is critical mass and momentum behind HIV vaccine R&D in the EU. The lack of supranational coordination and of a global HIV vaccine strategy is hindering progress in HIV vaccine research. With many competing health priorities, unresolved vaccine design challenges, diverse vaccine strategies, and limited funding, a more coordinated and gated approach to vaccine R&D is needed at European level.

Complementarity exists among various infectious diseases, and while it is plausible to adopt a more holistic approach to vaccine research, the efficacy of coordinated programmes lies in their ability to unite stakeholders and concentrate efforts on a singular question that can be addressed only through large collaborative consortia. Although challenging endeavours, past and current consortia have shown that European researchers, clinicians, and affected communities can work together and deliver high-quality science with limited funding.

Political commitment is necessary for progressing HIV vaccine development, especially in Europe where the research ecosystem is complex and not well known or understood. The UK rejoining Horizon Europe under a new bespoke deal is a positive step forward. Effective communication with decision-makers is key to securing funding for HIV vaccine research in Europe. Research needs to be positioned within existing national and supranational structures in Europe, as well as maintaining and creating linkages with other players in the field while emphasising the positive impact of HIV R&D on the prevention of other diseases.

Europe can lead on the construction of a sustainable R&D ecosystem to accelerate the transition from research to development and biomanufacturing, overcoming the “valleys of death” that hinder product development. The creation of a European R&D integrated end-to-end product development strategy building on current EC and national funding initiatives, each playing a well-defined role in product development, would streamline product development, avoid duplication of efforts, and strengthen a collaborative approach. A product portfolio approach aligned with a roadmap similar to those developed for other infectious diseases (WHO R&D blueprints and the Tuberculosis vaccine roadmap supported by EDCTP) would position individual efforts within a cohesive research continuum and enable developing multiple candidates simultaneously with the prospect of taking forward the most promising products. The pursuit of an HIV vaccine must be integrated into a comprehensive plan to enhance the prospects of securing diverse and robust funding, facilitate the establishment of necessary public-private research partnerships and ensure a seamless transition between funding phases to prevent capital loss. Such plan could itself be part of a global cross-cutting approach to tackle infectious diseases.

## Conclusion

Millions of people in the WHO European Region continue to experience the negative impact of HIV on their health and well-being. Current treatment and prevention will most likely not be enough to control the epidemic, let alone end it. Perception of HIV vaccine R&D, its relevance, and the tension between PrEP, long-term treatments and vaccines remain obstacles to successfully engaging with stakeholders and decision-makers at national and European levels. The perception that one size will fit all needs to be addressed since a range of complementary prevention and treatment options and tools are needed. Increased efforts must be made to showcase the achievements and benefits of European HIV vaccine research and to effectively convey the message that the HIV/AIDS epidemic is not over, that an HIV vaccine is still crucially needed and that it will bring long-term benefits including saving lives.

Promising vaccine candidates are being developed in Europe but are at risk without the long-term and sustained investments necessary to safeguard their development. The value of ongoing R&D for an HIV vaccine remains undiminished. Excitement and novelty can overcome “HIV fatigue” and help focus on solutions and plans for HIV R&D. Renewed energy is sparked by new opportunities for innovation that leverage new vector technology and novel immunological targets, as well as big data and artificial intelligence. There is an urgent need for comprehensive programmes and frameworks that facilitate the transition of academic and biotech innovations to industry. The response to COVID-19 has begun to shape the landscape for a bold Europe-led vaccine development programme which must include HIV. Failing to actively participate in HIV vaccine R&D amounts to renouncing the commitment to fulfill Sustainable Development Goal 3, which aims to ensure healthy lives and promote well-being for all at all ages. The EC and Europe can play a leadership role in establishing a research ecosystem that contributes to global health improvement and drives economic and social development. With the backing of a political message from the Member States, this collective effort can foster and reinforce multidisciplinary and multi-sectoral collaboration to establish and execute a comprehensive European strategy for the development of an HIV vaccine.

## Supporting information

S1 Table HIV vaccine EC-funded HIV vaccine research consortia that undertook the development and clinical testing of HIV vaccine candidates (Capacity-building and fellowship programmes are not included).
(DOCX)
