# Supplementary material for: In danger: HIV vaccine research and development in Europe
Source: PLOS Glob Public Health. 2025 Apr 8;5(4):e0004364. doi: 10.1371/journal.pgph.0004364 (PMC11977976; doi:10.1371/journal.pgph.0004364)
Supplement: S1 Table — HIV vaccine EC-funded HIV vaccine research consortia that undertook the development and clinical testing of HIV vaccine candidates (Capacity-building and fellowship programmes are not included). (DOCX) [file pgph.0004364.s001.docx]

| Programme | Funder | Description | Year | Funding |
| --- | --- | --- | --- | --- |
| EUROVAC I | FP5 | EuroVacc’s core activity is to facilitate the development of vaccine candidates through early clinical trials, to enable early identification of the promising vaccine candidates and eliminate candidates unlikely to succeed in the clinic, with the ultimate goal to reduce the development risks and accelerate the advancement of the promising candidates to large clinical trials.  https://cordis.europa.eu/project/id/QLK2-CT-1999-01321 | 2000-2005 | Total  €15.2M  EC  €8.8M |
| EUROVAC II | FP6 | The objectives of the EuroVac- II project are to demonstrate in a phase I trial of humans: firstly, the ability of Semliki Forest virus (SFV) to prime anti-HIV immune responses, - compared to the attenuated poxvirus NYVAC -, using a recombinant gp140 protein boost; and secondly the ability of SFV priming, - using DNA priming as benchmark -, to improve the immune responses elicited by NYVAC + rgp140 vaccination. https://cordis.europa.eu/project/id/QLK2-CT-2001-01316 | 2001-2005 | Total  €4.9M  EC  €3M |
| EUROVAC III | FP6 | The objective of the Euro Vac-III demonstration project was to identify the best priming agent, DNA vs. SFV, that allows for reduction of the NYVAC dose without significant loss of immunogenicity.  https://cordis.europa.eu/project/id/QLK2-CT-2002-01431 | 2002-2007 | Total  €3.9M  EC  €2.5M |
| HIVAB | FP6 | The objective of this project was to generate broadly cross neutralising Antibodies for innovative active-passive HIV Vaccination Strategies based on modified Ig-gene transgenic mice  https://cordis.europa.eu/project/id/19052 | 2005-2008 | Total  €1M  EC  €0.9M |
| Afrevacc | EDCTP 1 | This multi-centre international project aimed to develop a comprehensive network of institutional partners from Europe and Africa and to use their existing data and expertise to build new capacity for HIV vaccine trials in Mozambique, South Africa, and Tanzania.  https://www.edctp.org/annualreport2011/clinicaltrials/03_weber.html | 2008-2012 | Total  €6.9M  EDCTP  €3.3M |
| AFO-18 | EDCTP 1 | This was a joint initiative to sustain HIV vaccine trials and research capacity in the Republic of Guinea-Bissau, West Africa.  https://www.edctp.org/annualreport2010/grants_2010/fomsgaard.html | 2010-2012 | Total  €2 M  EDCTP  €231,859 |
| PedVacc | EDCTP 1 | The aim of this study was to develop a vaccine for prevention of vertical transmission of HIV-1 during breastfeeding.  https://www.edctp.org/annualreport2011/clinicaltrials/10_hanke.html | 2008-2012 | Total  €3.3M  EDCTP  €3M |
| HIV-CORE 004 | EDCTP 1 | This was a Phase I/IIa clinical trial of universal HIV-1 vaccines pSG2.HIVconsv, MVA.HIVconsv, and Ad35-GRIN.HIVconsv in combined regimens in healthy HIV-1/2-negative adults in Nairobi, Kenya. | 2012-2015 | Total  €1.2M  EDCTP  €0.8M |
| TAMOVAC I | EDCTP 1 | This Phase I/II trial assessed safety and immunogenicity of intradermal (ID) DNA priming, intramuscular (IM) MVA, and IM rgp140/GLA-AF boosting in healthy volunteers in Tanzania and aimed to develop further HIV vaccine trial capacity building in Tanzania.  https://www.edctp.org/annualreport2010/clinical trials/05_bakari.html | 2008-2012 | Total  €6.8M  EDCTP  €3.3M |
| TAMOVAC II | EDCTP 1 | The project continued exploring optimal DNA-priming and MVA-boosting strategies developed under TAMOVAC I.  https://www.gesundheitsforschung-bmbf.de/en/hiv-vaccines-tamovac-ii-evaulation-of-the-safety-and-the-immune-response-after-hiv-7693.php | 2009-2015 | €779,815 |
| CUT'HIVAC | EU – FP7 | This collaborative effort involved 13 partners from five European countries and two International Cooperation countries. Its primary goal was to tackle the challenge of developing a new strategy for responding to HIV by leveraging expertise in vaccinology and HIV diseases. The consortium aimed to achieve this by adopting an innovative approach that utilises transcutaneous and/or mucosal needle-free vaccination methods.  https://cordis.europa.eu/project/id/241904 | 2010-2015 | €15.4M |
| MucoVac | EU – FP7 | The programme focused on: (i) performing innovative research on mucosal adjuvants’ mode of action; and (ii) catalysing the mid-term development of novel, safe, efficient vaccines for nasal or oral administration.  https://cordis.europa.eu/project/id/657107 | 2016-2018 | €185.076 |
| EUROPRISE | EU – FP7 | This was the first organization in Europe and internationally to purposely bring vaccine and microbicide research groups together in a truly integrated fashion, promoting a joint programme of research, from early discovery through to early clinical trials.  https://cordis.europa.eu/project/id/37611/fr | 2007-2012 | €15.5M |
| EURONEUT-41 | EU – FP7 | The EUROpean consortium on NEUTralising antibodies using gp41 (EuroNeut-41) project involves 17 partners and had for overall objective the design of a vaccine that raises antibodies able to prevent HIV cell fusion by blocking the virus fusion protein gp41 in its pre-fusogenic conformation.  https://cordis.europa.eu/project/id/201038/reporting | 2008-2014 | €15.9M |
| EAVI 2020 | H2020 | The European AIDS Vaccine Initiative 2020 brought together leading HIV researchers from public organisations and biotech companies in Europe, Australia, Canada, and the USA in a focused effort to develop protective and therapeutic HIV vaccines. EAVI 2020 united scientists from 22 institutions to develop novel candidate vaccines that can be taken through to human trials within five years.  https://cordis.europa.eu/project/id/681137 | 2016-2023 | €24.1M |
| EHVA | H2020 | EHVA brought together 41 partners to promote a comprehensive approach to the development of an effective HIV vaccine. The alliance included academic and industrial research partners from all over Europe, sub-Saharan Africa, and North America to work towards discovering and progressing novel vaccine candidates through the clinic.  https://cordis.europa.eu/project/id/681032 | 2016-2023 | €28.2M |
| GREAT | EDCTP 2 | The collaboration between institutions in Europe and Africa is advancing the development of a promising global preventive HIV-1 vaccine strategy. The working hypothesis is that focusing HIV-specific killer T cells on the most conserved regions of the HIV proteome will lead to control of HIV replication.  https://publications.edctp.org/international-partnerships-against-infectious-diseases/great | 2017-ongoing | Total  €16.8M  EDCTP  €7.1M |
| PrEPVacc | EDCTP 2 | This Phase IIb, three-arm, two-stage HIV vaccine efficacy trial with a second randomisation to compare the newer PrEP drug Descovy with the established standard for PrEP, Truvada.  https://publications.edctp.org/international-partnerships-against-infectious-diseases/prepvacc | 2018-ongoing | Total  €22.2M  EDCTP  €15M |
| CAP012 SAMBA | EDCTP 2 | The project builds on the discovery of a broadly neutralising antibody, known as CAP2456-VRC26.25, in a woman from KwaZulu-Natal, South Africa. The CAP012 SAMBA is analysing the safety of this and two other antibodies in people, exploring their acceptability to potential recipients, and monitoring their metabolism in the body. On the basis of these studies, the most promising combinations of antibodies will be taken forward to a Phase II trial to assess safety and efficacy in preventing HIV acquisition.  https://publications.edctp.org/international-partnerships-against-infectious-diseases/cap012-samba-trial | 2018- ongoing | Total  €18.7M  EDCTP  €9.3M |
| Neo bnAB | EDCTP 2 | The study is focused on one specific broadly neutralising antibody known as VRC01. Minor modifications have created a slightly different version, VRC01LS, that survives longer in the bloodstream, so could potentially provide protection for longer periods and require less frequent administration. Initial studies have shown that VRC01LS is safe to give to newborns.  https://publications.edctp.org/international-partnerships-against-infectious-diseases/neo-bnab | 2019-ongoing | Total  €4.8M  EDCTP  €4·2M |
| PedMAb | EDCTP 2 | PedMAb’s aim is to ensure the development of a promising HIV-MTCT prevention strategy with broad anti-HIV-1 neutralizing monoclonal antibodies (bNAbs). The main objective is to complete two clinical trials, to define the optimal dose(s), timing, and the ideal combination(s) of bNAbs administered to breastfeeding HIV exposed uninfected neonates and infants born to HIV-infected women. | 2021-ongoing | EDCTP  €3.9M |
